# Pulse Wave Velocity and Sarcopenia in Older Persons—A Systematic Review and Meta-Analysis

**DOI:** 10.3390/ijerph19116477

**Published:** 2022-05-26

**Authors:** Karolina Piotrowicz, Alicja Klich-Rączka, Anna Skalska, Barbara Gryglewska, Tomasz Grodzicki, Jerzy Gąsowski

**Affiliations:** Department of Internal Medicine and Gerontology, Jagiellonian University Medical College, 2 Jakubowskiego St., 30-668 Krakow, Poland; karolina.piotrowicz@uj.edu.pl (K.P.); alicja.klich-raczka@uj.edu.pl (A.K.-R.); anna.skalska@uj.edu.pl (A.S.); barbara.gryglewska@uj.edu.pl (B.G.); tomasz.grodzicki@uj.edu.pl (T.G.)

**Keywords:** arterial stiffness, sarcopenia, meta-analysis, PWV

## Abstract

Sarcopenia and cardiovascular disease share some of the pathophysiologic mechanisms. Sarcopenia is likewise an important feature of frailty and the one potentially related to cardiovascular pathology. Previously, the relationship between arterial stiffness and frailty has been established. In this study, we conducted a systematic review and a meta-analysis of studies where the relationship between pulse wave velocity (PWV) and sarcopenia has been addressed. We included six cross-sectional studies that enrolled 5476 participants. Using the WebPlotDigitizer, RevMan5, and SAS 9.4, we extracted or calculated the summary statistics. We then calculated standardized mean differences (SMD) of PWV in the sarcopenic and non-sarcopenic participants. The pooled SMD was 0.73 (95% CI 0.39–1.08, *p* < 0.0001, I^2^ = 90%) indicating higher value in the sarcopenic subjects. The three studies that presented odds ratios for sarcopenia as a function of PWV homogenously indicated a greater probability of concomitant sarcopenia with higher values of PWV. Greater stiffness of the aorta is associated with sarcopenia. It is impossible to establish the causation. However, the plausible explanation is that increased stiffness may translate into or be an intermediary phenotype of common vascular and muscle damage. On the other hand, sarcopenia, which shares some of the inflammatory mechanisms with cardiovascular disease, may wind up the age-related large arterial remodeling.

## 1. Introduction

Sarcopenia, defined as decreased muscle mass, deranged muscle architecture and decreased muscle strength, is an important feature of unfavorable aging [1]. It has been linked with worse survival, poor functionality and greater cardiovascular risk [2,3]. In a broader context, many older persons with frailty, at the core of the general deterioration have sarcopenia [4].

Large arterial stiffening is one of the hallmarks of aging of the cardiovascular system [5]. When advanced, such stiffening starts to be associated with a number of pathologies, among them isolated systolic hypertension, the predominant form of hypertension in older adults [6,7]. Of the measures of vascular stiffness, pulse wave velocity (PWV), especially measured over the distance between carotid and femoral arteries, has been linked with an increased risk of cardiovascular complications and mortality [8]. It is likewise possible that vascular damage described by higher PWV may impact skeletal muscular mass, structure and function, probably acting via impediment to muscular circulation [9,10]. On the pathophysiologic plane, a low-grade inflammation, sometimes referred to as inflammaging, insulin resistance and hyperinsulinemia, mitochondrial dysfunction and oxidative stress are examples of the potential link between sarcopenia and large arterial remodeling [10,11,12,13].

Previously, we reviewed the available literature linking large arterial stiffness with frailty [14]. We found that greater values of the indices of stiffness such as pulse wave velocity or the cardio-ankle vascular index (CAVI) bear with them a higher probability of concomitant frailty. Likewise, frail persons have higher values of stiffness indices [14,15]. Several studies and narrative reviews focused on sarcopenia and vascular damage [15,16,17]. However, the question of whether the components of frailty, such as sarcopenia, would themselves be linked to greater stiffness has not been comprehensively answered. While some of the studies showed a relation between sarcopenia and arterial stiffness, others were far from conclusive. Additionally, the methodology of the studies, both when it comes to vascular and muscular assessment, differed. Likewise, a number of studies and meta-analyses addressed the issue of the relationship between skeletal muscle mass and arterial stiffness [18]. However, muscle mass is but one aspect of possible sarcopenia. Accordingly, to draw broader conclusions, a common discussion of such studies trying to place them on a common denominator seemed warranted. In the present review and meta-analysis, our aim was to check whether sarcopenia is associated with greater PWV. We, therefore, bring together published evidence linking greater PWV to sarcopenia in older adults.

## 2. Materials and Methods

The methodology we used, and hence its description, mirrors the one we previously used for the reference [14].

Between the 20th and 23rd of March 2022, we searched the PubMed, Web of Science, and EMBASE with the following terms:

*(sarcopenia [Title/Abstract]) AND (arterial stiffness [Title/Abstract]); (sarcopenia [Title/Abstract]) AND (cardio-ankle vascular index [Title/Abstract]); (sarcopenia [Title/Abstract]) AND (cavi [Title/Abstract]); (sarcopenia [Title/Abstract]) AND (pulse wave velocity [Title/Abstract]); (sarcopenia [Title/Abstract]) AND (pwv [Title/Abstract]); (sarcopenia [Title/Abstract]) AND (augmentation index [Title/Abstract]); (sarcopenia [Title/Abstract]) AND (aIx [Title/Abstract])*

**Figure 1:**
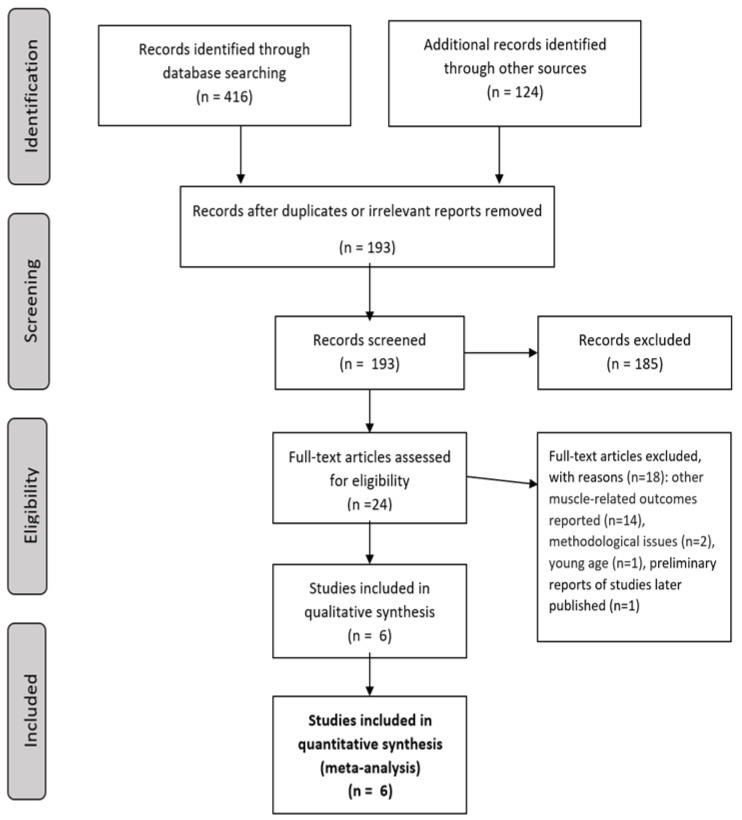
The PRISM diagram for the review and the meta-analysis.

We additionally searched the reference lists of the retrieved manuscripts and checked the manuscripts citing the retrieved papers. We especially searched through the reference lists of previous narrative reviews of the topic. Figure 1 presents the flow of the inclusion of studies in the present review.

The review was performed according to the systematic reviews and meta-analysis (PRISMA) guidelines [19,20]. The literature search and manuscript selection were performed by two independent researchers (KP, AS). The data extraction was performed by KP and JG with the help of AS. The quality of the evidence in respective manuscripts was rated according to the Joanna Briggs Institute criteria for cross-sectional studies [21]. We limited our search to the English language reports of human studies.

In some studies, the standard errors of the mean or standard deviations had to be recalculated based on the available data, or the between-group t-statistic or chi-square had to be calculated. In the case of one paper [22], the approximate data were extracted with the use of the web-based WebPlotDigitizer (https://apps.automeris.io/wpd/, accessed on 22 March 2022, Oakland, CA, USA) software.

Based on the data from three studies [23,24,25], it was possible to obtain odds ratios for sarcopenia concomitantly associated with 1-unit-higher PWV. For the analysis based on mean values of PWV measures, due to the fact that they were obtained with the use of two different modalities, aortic-brachial PWV and the brachial-ankle PWV (baPWV), we calculated standardized mean differences with 95% CI. The standardized mean differences (SMD) enable comparison of the results from different modalities by putting them on the standard deviation scale. The pooled analyses were performed with random models, using weighing by the inverse of the variance. For each type of analysis, we obtained the I^2^, a measure of heterogeneity. The statistical calculations were performed with the RevMan5 (Copenhagen, Denmark), SAS 9.4 (Cary, NC, USA) or pocket calculator, as applicable. The *p*-value of <0.05 was considered significant.

## 3. Results

### 3.1. The Characteristics of the Included Studies

Of the initial 540 abstracts, in the analysis, we included six cross-sectional studies (Figure 1), with a total of 5476 participants aged above 60 years. The average age (standard deviation) of participants in five studies where data were available for sarcopenic and non-sarcopenic participants was 70.2 (4.9) years. In total, 51.8% of participants in these studies were male. The study by Ohara et al., apart from the included sarcopenic and non-sarcopenic participants, included groups with obesity and sarcopenic obesity, which were not included in our analyses as they might bias the relation between PWV and sarcopenia. Based on the report by Ohara et al., it was impossible to extract the information concerning age and gender distribution in the sarcopenic and non-sarcopenic patients (Table 1) [22].

The study by Öztorun et al. included 72 older geriatric clinic outpatients (Ankara City Hospital, Ankara, Turkey) who fell at least once during the year preceding enrolment [23]. For the analyses, we used data from 12 persons without sarcopenia and 28 persons with sarcopenia. The mean (standard deviation) age was 79.2 (6.4) years. The patients with sarcopenia were significantly older, had less hypertension, were more often smokers and were leaner (all *p* ≤ 0.03) (Table 1). Sun et al. included 2511 older community dwellers aged ≥60 years [26]. The mean age was 68.6 (5.8) years. Blood pressure values were not reported. Compared with non-sarcopenic participants, the sarcopenic persons were older, were more often hypertensive, diabetic and had more cardiovascular complications in the past (all *p* ≤ 0.002). The study by Rong et al. [24] included 450 participants. The study was performed in the Health Management Centre of Tianjin First Central Hospital, China. The average age was 71.3 (4.2) years. The sarcopenic patients were older and leaner (all *p* ≤ 0.005). There were no differences between the sarcopenic and non-sarcopenic participants with respect to hypertension, cardiovascular disease, diabetes and smoking status. Zhang Y. et al. included 1046 community-dwelling older persons as participants of the Wakayama Health Promotion Study [27]. We included data from 746 persons with (70) and without sarcopenia. The mean age was 71.4 (4.3) years. The sarcopenic persons were older and leaner; however, they smoked less often. There was no difference in blood pressure (Table 1). Zhang L. et al. included 1002 community-dwelling persons ≥65 years of age. The mean age was 72.3 (5.2) years. On top of data on baPWV, no other data were available in the subgroups of sarcopenic and non-sarcopenic patients [25]. Ohara et al. included data from 1593 persons. In the analysis where sarcopenia was based on the handgrip strength assessment, the summary data of 463 persons were available, whereas when sarcopenia was defined based on skeletal muscle mass, summary data of 727 persons were available. On top of data on baPWV, no other data were available for the subgroups with and without sarcopenia [22].

### 3.2. The Measures of Sarcopenia

The studies by Rong et al. and Zhang L et al. used the Asian Working Group for Sarcopenia (AWGS) 2014 definition [24,25]. The study by Zhang Y et al. used two of the three AWGS 2014 criteria, namely hang grip strength and appendicular skeletal muscle mass (ASM); however, for the SMA, they used their own threshold (Table 2) [27]. Sun et al. used the current AWGS 2019 criteria [26]. Öztorun et al. used the European Working Group on Sarcopenia in Older People 2 (EWGSOP2) criteria with national modification of hand-grip strength cut-offs (Table 2) [23,28]. Ohara et al. based their assessment on either the handgrip strength or skeletal muscle mass (SMM). They defined sarcopenia as handgrip strength or SMM lower than the lower 1 SD for the population aged 50 years. Alternatively, they diagnosed sarcopenia when handgrip strength or SMM was in the lowest quintile of the distribution [22]. For our analyses, we used the data based on the standard deviation principle, which were compatible with the concept of sarcopenia.

### 3.3. Pulse Wave Velocity

The study by Öztorun et al. used the Mobil-O-Graph cuff-based methodology, which estimates the PWV in the aortic-brachial segment (Table 2) [23]. The studies by Sun et al., Zhang Y et al., Zhang L et al., Rong et al. and Ohara et al. used the brachial-ankle cuff-based PWV assessment (Table 2), where the waveforms from the arm and calf are obtained with plethysmographic method [22,24,25,26,27]. However, the equipment used was different in each case (Table 2).

### 3.4. Relation between PWV and Sarcopenia

**Figure 2:**
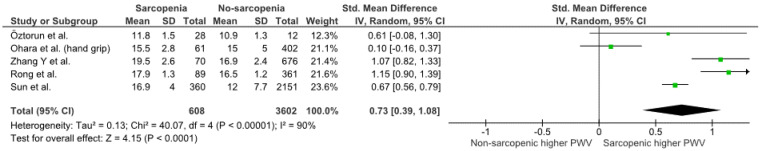
The within-study means and standard deviations of the within-group pulse wave velocity with within-study and pooled standardized mean differences (SMD) [22,23,24,26,27]. Handgrip-based definition of sarcopenia for Ohara et al.’s study [22], The green squates are Std. Mean Differences as stated above the graphs.

**Figure 3:**
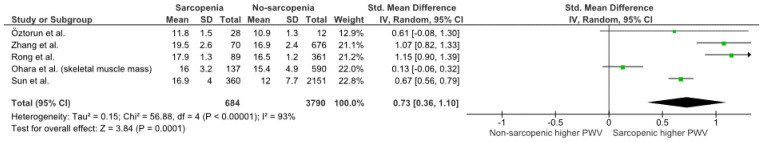
The within-study means and standard deviations of the within-group pulse wave velocity with within-study and pooled standardized mean differences (SMD) [22,23,24,26,27]. Skeletal-muscle-mass-based definition of sarcopenia for Ohara et al.’s study [22].

**Figure 4:**
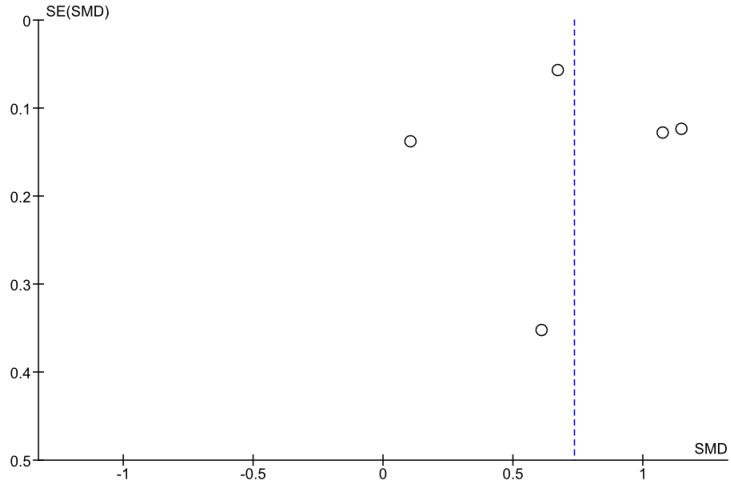
The funnel plot for the studies from Figure 2.

**Figure 5:**
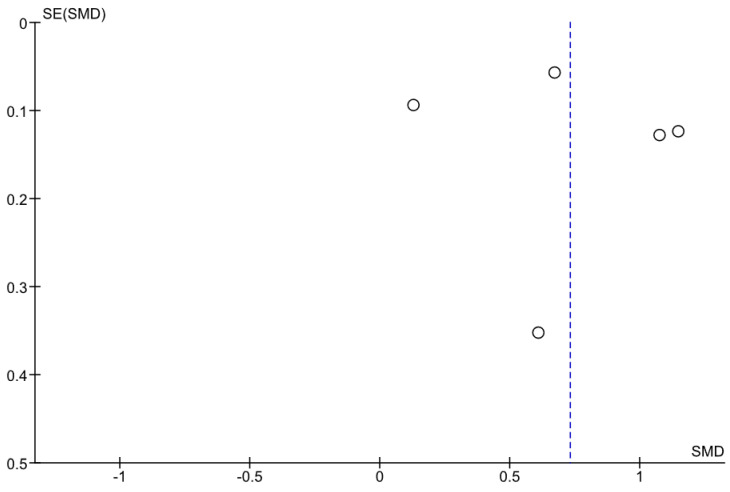
The funnel plot for the studies from Figure 3.

Five studies included retrievable information about average PWV in sarcopenic and non-sarcopenic participants. In the studies by Sun et al., Rong et al. and Zhang Y. et al., the PWV values in sarcopenic participants were greater than in the non-sarcopenic ones (all *p* < 0.0001, Figure 2) [24,26,27]. In the remaining studies, the point estimates for the standardized mean difference were pointing toward higher PWV values in sarcopenic groups. In the sensitivity analysis, where the data on muscle mass rather than handgrip from the study by Ohara et al. were used, the results were not materially different (Figure 3) [22]. The heterogeneity as measured with I^2^ ≥ 90% (Figure 2, Figure 3, Figure 4 and Figure 5).

In three studies (Öztorun et al., Rong et al., Zhang L. et al.), the authors presented the odds ratios for sarcopenia given the PWV [23,24,25]. All these studies indicated a significantly greater probability of concomitant sarcopenia in persons with greater PWV (all *p* < 0.04, Table 3). In the study by Öztorun et al., the significance of the relation was lost when PWV was put into one model with age and hypertension [23]. In that study, the model in which PWV displayed the significant relation was adjusted for daytime systolic blood pressure, nighttime mean arterial pressure and heart rate, daytime pulse pressure and daytime peripheral resistance [23]. In the study by Zhang L. et al., the model was adjusted for age, gender and BMI. Additional adjustment for history of stroke did not materially alter the results [25]. In that study, the authors give the odds ratio associated with 1SD (that is 3.5 cm/s) greater PWV [25]. In the study by Rong et al., the model was adjusted for age, body mass index, sports activity, visceral fat and the mini nutritional assessment short-form results [24].

## 4. Discussion

Based on the published data, we found that brachial artery PWV is consistently higher in older persons with sarcopenia than in persons free from the condition. We also found that the odds of concomitantly being sarcopenic are higher in persons with higher baPWV.

### 4.1. Sarcopenia and Cardiovascular Disease

Sarcopenia, an important outcome measure in older persons, has been linked to cardiovascular risk factors and cardiovascular disease, hence the importance of elucidating the factors, especially those potentially amenable to therapy, that in the pathology of older persons might be implicated in its emergence. The link between sarcopenia and cardiovascular risk factors or atherosclerotic cardiovascular disease is probably bi-directional. First, from hypertension, through diabetes mellitus, to established atherosclerotic cardiovascular disease and complications, the patients affected have more muscular damage [3,34,35,36]. On the other hand, sarcopenia mapped to wound-up inflammaging has been considered a factor in vascular damage [15].

### 4.2. Aging of Arterial System

Aging is associated with physiological vascular remodeling. The factors implicated in the remodeling include physical wear-and-tear after cumulative billions of heartbeats over the lifetime, but also low-grade inflammation acting in part via the endothelial dysfunction [5].

One of the facets of age-related vascular changes is the stiffening of the arterial tree, resulting in the faster propagation of the forward and reflected wave of the pressure pulse wave. This can be measured using several modalities such as aortic or regional pulse wave velocity [37,38]. The increased PWV has, in turn, been bi-directionally related to a wide range of cardiovascular risk factors and cardiovascular complications [39]. This has been especially pronounced in older persons or in persons with concomitant conditions, such as chronic kidney disease, which can be considered models of premature vascular aging [40,41].

### 4.3. Putative Pathophysiologic Link between Arterial Stiffness and Sarcopenia

None of the studies we included was designed to answer the question of causality, nor is it possible to address that issue based on the results of our meta-analysis. This is primarily due to the cross-sectional nature of the included studies. However, the influence is likely to be bi-directional. The age-related and pathology-augmented loss of muscle mass, worsening of muscular architecture and resulting declining performance may impact vascular biology. The possible mechanisms would include insulin resistance and resulting hyperinsulinemia that, via the trophic effect of arterial wall components, may increase the wall remodeling and especially lead to an increase in wall thickness and loss of elastic properties [10,42]. Sarcopenia has been related to increased inflammation. It has been shown that elevation of IL-6 and CRP especially play a role as a possible inflammatory link between sarcopenia and cardiovascular risk [11]. All of this may lead to higher systolic blood pressure (SBP), greater pulse pressure (PP) and faster propagation of pressure pulse wave. This, in turn, has been shown to increase cardiovascular risk in older subjects [3,6,43].

On the other hand, the age-related remodeling of large arteries and the life-long exposure to other cardiovascular risk factors may lead to a worsening of muscular blood supply and increased muscular oxidative stress, both leading to metabolic imbalance and muscular wasting [44]. Of note, insulin resistance has been implicated in increasing oxidative stress, which creates a pathophysiologic vicious circle [45]. In the cases of clinically overt coronary artery disease, peripheral artery disease and heart failure, the symptom-induced inactivity might further decrease the stimuli for muscular growth.

The results of our analysis and the conclusions that can be drawn from it heavily depend on the methodological aspects of the included studies.

### 4.4. The Impact of Methodology of PWV Assessment on the Meta-Analysis Results

First, the methods for the assessment of vascular stiffness were not homogenous. None of the studies used the gold-standard method of direct ECG-gated carotid-femoral pressure pulse wave measurement [46]. Whereas in five out of six studies, the brachial-ankle cuff-based method to estimate the PWV was used, one study by Öztorun et al. used the method based on a single cuff placed over the brachial artery (Table 2) [23].

The cuff-based PWV methodology has been charged with greater dependence on age and blood-pressure level [38,47]. It is also dependent not only on the status of large conduit arteries (predominantly aorta) but also on the status of muscular arteries (brachial and tibial) [48,49]. The remodeling in the elastic and muscular arteries differs, as does the influence of risk factors on tibial and brachial arteries. Whereas in the former, atherosclerosis is common, it is rarely seen in the latter [50]. Likewise, the distance between the measurement points is further apart. Finally, it is possible that the direct influence of already ongoing sarcopenia on muscular arteries supplying the muscles may further confound the PWV and its relationship with sarcopenia. All this may have influenced the results and the relations between the PWV and outcome, sarcopenia in our case. In one study (Table 2), the Mobil-O-Graph methodology was used, which in principle gives an estimated value of PWV over the aortic-brachial segment [23]. In order to overcome the variability of measurement methods, we used the standardized mean differences that present the between-group differences as the standard deviation units [51].

### 4.5. The Impact of Methodology of Muscular Assessment on the Meta-Analysis Results

The methodology of muscular assessment differs considerably between studies and largely deviates from the current comprehensive approach to the diagnosis of sarcopenia (EWGSOP2, AWGs) (Table 2) [29,31]. One study used the recent EWGSOP2 criteria; however, the authors modified the cut-off values for the handgrip by including the values validated for their national population and published elsewhere (Table 2) [23,28]. Another study used the current AWGS criteria for the diagnosis of sarcopenia [26]. In three studies, the authors used the older AWGS definition; however, in one of these studies, the cut-off values for ASM/ht2 were modified with values derived from the population in which the study was performed, i.e., sex-specific lowest quintile value derived from the study population (Table 2) [24,25,27]. One study used two different definitions for the handgrip cut-off, namely <1 standard deviation of the value for the population of persons < 50 years of age or <20% of the sex-specific values derived from the population under study [22]. In this study, a similar approach was adopted for the muscle mass cut-off values. In line with older definitions of sarcopenia, we used the data based on the 1SD principle. Additionally, to diagnose sarcopenia, the authors used either a handgrip-strength- or muscle-mass-based approach [22]. The bioimpedance equipment used was heterogeneous, as were the formulas used to estimate the muscle mass (Table 2). The differences in the definitions and the assessment of the outcome may have inflated the heterogeneity of the overall estimate, with the I^2^ in excess of 90%.

Additionally, the populations included in the analyzed studies differed significantly with respect to whether they were hospitalized or not, were healthy or burdened with diseases, or were younger or older (Table 1).

### 4.6. Limitations

Our analysis needs to be taken in the context of its limitations. First, as mentioned above, there were numerous sources of possible heterogeneity. These included the varying definitions of sarcopenia and two different methods to assess PWV. However, the point estimate values of the standardized mean difference of PWV were in all studies pointing to higher values in sarcopenic persons. Second, it was impossible to address the issue of causality. However, based on the current pathophysiologic understanding, the relation is likely to be bi-directional, assuming a form of a vicious circle. In our analyses, we decided not to consider sarcopenic obesity, as it might obscure the relation between sarcopenia and cardiovascular parameters such as arterial stiffness. Additionally, most of the studies that we included did not include the data on sarcopenic obesity. Some of the participants of the included studies may have had sarcopenic obesity. Further, in the studies where the authors modeled the risk of concomitant sarcopenia as a function of PWV, the adjustment of the models varied between the studies, and only one of three studies clearly stated the amount of increment of PWV for which the odds ratio was presented [25].

## 5. Conclusions

In conclusion, there is a definite link between the stiffening of large conduit arteries and sarcopenia, and the associations are likely to be mutually winding up. More advanced vascular aging adversely impacts skeletal muscles, while the decline of muscular mass and strength may, in turn, add to the cardiovascular burden. From the practical standpoint, any older patient with stiff arteries should be assessed on account of possible sarcopenia, while an overtly sarcopenic person should have their arterial stiffness assessed. The presence of both sarcopenia and elevated PWV might indicate increased cardiometabolic risk, which should warrant a thorough assessment of other risk factors. This could lead to a step up in both non-pharmacological and pharmacological therapy. In order for any of this to be meaningful, a unified definition of sarcopenia, taking into account its pathophysiologic complexity (EWGSOP2, AWGS2), should be used. On the other hand, methods such as the carotid-femoral PWV, either directly measured or inferred based on the waveform analysis, should be preferred over other methods [46,52].

## Figures and Tables

**Table 1:** The characteristics of the included studies.

Study	*n*	Male Sex (%)	Age (SD), Years	Non-Sarcopenic (%)	Age (SD), Years	SBP(SD)/DBP(SD) mm Hg	BMI (kg/m^2^)	HT (%)	CAD (%)	DM (%)	Ever Smoking (%)	Sarcopenic (%)	Age (SD), Years	SBP(SD)/DBP(SD) mm Hg	BMI (kg/m^2^)	HT (%)	CAD (%)	DM (%)	Ever Smoking (%)
Öztorun et al. 2022 [23]	40	30.0	79.2 (6.4)	30.0	75.6 (5.4) (**p* =* 0.03)	142.4 (21.6)/ 79(65–93) (*p* = 0.17/…)	29.4 (5.0) (*p* = 0.003)	91.7 (*p* = 0.02)	33.3 (p= 0.72)	66.7 (*p* = 0.11)	50.0 (*p* = 0.02)	70.0	80.7 (6.8)	131.3 (23.5)/ 70(60–111)	23.9 (4.8)	53.6	39.3	39.3	85.7
Sun et al. 2021 [26]	2511	41.3	68.6 (5.8)	85.7	68.4 (5.7) (*p* = 0.006)	…	24.4 (3.3) (*p* = 0.60)	35.5 (*p* = 0.0004)	10.5 (*p* = 0.002)	12.8 (*p* = 0.0001)	…	14.3	69.3 (6.0)	…	24.5 (3.4)	45.3	16.1	20.3	…
Rong et al. 2020 [24]	450	59.1	71.3 (4.2)	80.2	71.1 (4.2) (*p* = 0.005)	…	25.2 (2.3) (*p* = 0.000)	29.1 (*p* = 0.66)	28.0 (*p* = 0.29)	13.6 (*p* = 0.60)	13.6 (*p* = 0.43)	19.8	72.5 (4.7)	…	24.0 (1.9)	31.5	33.7	15.7	16.9
Zhang Y. et al. 2020 [27]	746	43.3	71.4 (4.3)	93.3	71.1 (4.2) (*p* < 0.0001)	131 (17)/ 75(10) (*p* = 1.0/0.42)	23.8 (3.0) (*p* < 0.0001)	…	…	…	33.6 (*p* = 0.04)	6.7	74.1 (5.6)	131 (19)/74 (9)	19.6 (2.4)	…	…	…	21.4
Zhang L. et al. 2019 [25]	1002	41.9	72.3 (5.2)	89.3	…	…	…	…	…	…	…	10.7	…	…	…	…	…	…	…
Ohara et al. 2015 [22]	463 * 727 **	…	…	86.8 * 81.2 **	…	…	…	…	…	…	…	13.2 * 18.8 **	…	…	…	…	…	…	…

*—Handgrip-based classification; **—skeletal muscle mass-based classification; SBP/DBP—systolic and diastolic blood pressure; BMI—body mass index; SD—standard deviation.

**Table 2:** The muscular and vascular methodology of the included studies.

	Sarcopenia Definition	Muscle Strength		Muscle Mass		Physical Performance		Arterial Stiffness
		Measure	Threshold	Measure	Threshold	Measure	Threshold	
Öztorun et al. 2022 [23]	EWGSOP2 criteria, 2018 [29]	Handgrip strength; electronic manual dynamometer (GRIP-D, Takei, Niigata, Japan) Dominant hand; the mean value of three measurements; done while the arm was in elbow flexion of 90°	Women < 22 kg Men < 32 kg	BIA Estimation of skeletal muscle mass (SMM) with Janssen formula [30] Absolute skeletal muscle mass (ASMM) = SMM/ht^2^	For ASMM/ht^2^: Women < 7.4 kg/m^2^ Men < 9.2 kg/m^2^	Gait speed (4 m)	<0.8 m/s	PWV (aorto-brachial) Mobil-O-Graph Blood Pressure 24 h PWA Monitor (IEM GmbH, Stolberg, Cologne, Germany)
Sun et al. 2021 [26]	AWGS criteria, 2019 [31]	Handgrip strength; a spring-type dynamometer (TSN100/WL, Physical Fitness Sports Technology Company, Beijing, China) Dominant hand; the maximum value of three measurements, conducted while standing and extending their arms at their sides	Women < 18 kg Men < 26 kg	BIA Estimation of skeletal muscle mass (SMM) with formula: SMM (kg) = 0.566 × FFM (fat free mass) [28] Appendicular muscle mass index (AMI) = appendicular skeletal muscle mass (ASM)/ht^2^	For ASM/ht^2^: Women < 5.7 kg/m^2^ Men < 7.0 kg/m^2^	…	…	baPWV IIM-AS-100 system (Institute of Intelligent Machines)
Rong et al. 2020 [24]	AWGS criteria, 2014 [32]	Handgrip strength; Jamar hand dynamometer 5030 J1 (Lafayette Instrument Company, Lafayette, IN, USA) Both hands; the max value of three measurements for each hand; conducted while sitting comfortably on a standard chair with legs, back support and fixed arms	Women < 18 kg Men < 26 kg	BIA (InBodyS10, InBody Japan Inc., Tokyo, Japan) Estimation of appendicular skeletal muscle mass (ASM) with Buckinx formula [33] Appendicular muscle mass index (ASMI) = appendicular skeletal muscle mass (ASM)/ht^2^	For ASM/ht^2^: Women < 5.7 kg/m^2^ Men < 7.0 kg/m^2^	Gait speed (6 m)	<0.8 m/s	baPWV VP1000 (Colin Company of Japan)
Zhang Y. et al. 2020 [27]	Sarcopenia: low HG + low ASM index	Handgrip strength; a Smedley type digital grip dynamometer (T.K.K.5401, TAKEI Scientific Instruments Co., Ltd., Niigata, Japan) Both hands; the max value of two measurements for each hand	Women < 18 kg Men < 26 kg	BIA (Physion MD, Physion Co., Ltd., Kyoto, Japan) The appendicular skeletal muscle mass (ASM) = the sum of skeletal muscle in the arms and legs ASM index = ASM/ht^2^	For ASM/ht^2^: Women < 3.7 kg/m^2^ Men < 4.4 kg/m^2^ (threshold modified by the researchers as sex-specific lowest quintile)	…	…	baPWV BP-203RPE II/III, Fukuda Colin Co., Ltd., Tokyo, Japan
Zhang L. et al. 2019 [25]	AWGS criteria, 2014 [32]	Handgrip strength Dominant hand; the mean value of two measurements	Women < 18 kg Men < 26 kg	BIA (InBody 770; Biospace Co., Ltd., Seoul, Korea) Appendicular skeletal muscle mass (ASM) = the sum of skeletal muscle in the arms and legs Relative skeletal muscle mass index =ASM/ht^2^	For ASM/ht^2^: Women < 5.7 kg/m^2^ Men < 7.0 kg/m^2^	Gait speed (4 m)	<0.8 m/s	baPWV Vascular Profiler-1000 device (Omron, Kyoto, Japan)
Ohara et al. 2015 [22]	Sarcopenia: -values of HG or SMM < −1SD of values obtained from patients aged below 50 years OR -values of HG or SMM in the lowest 20% of those obtained from all study participants of the same sex	Handgrip strength; (T.K.K. 5410; Takei Scientific Instruments Co., Ltd., Niigata, Japan) Both hands; the max value of one measurement for each hand	Values of HG < −1SD of values obtained from patients aged below 50 years OR values of HG in the lowest 20% of those obtained from all study participants of the same sex	BIA (HBF-701; Omron Healthcare Co. Ltd., Kyoto, Japan)	Values of SMM < −1SD of values obtained from patients aged below 50 years OR values SMM in the lowest 20% of those obtained from all study participants of the same sex	…	…	baPWV PWV/ABI; Omron Healthcare Co. Ltd.

SD—standard deviation; EWGSOP2—European Working Group on Sarcopenia in Older People 2 [29]; AWGS—Asian Working Group for Sarcopenia 2014 [32], 2019 [31]; BIA—bioimpedance analysis; SMM—skeletal muscle mass; AMS—appendicular muscle mass; ASMM—absolute skeletal muscle mass; FFM—fat-free mass; AMI—appendicular muscle mass index; ASM—appendicular skeletal muscle mass; ASMI—appendicular muscle mass index; PWV—pulse wave velocity, ba—brachial ankle; HG—handgrip; acronyms as given by the analyzed papers.

**Table 3:** Odds ratios of sarcopenia associated with PWV.

Study	OR of Sarcopenia Given 1 Unit Greater PWV	*p*-Value
Öztorun et al. 2021 [23]	1.98 (1.20–3.29)	0.008
Rong et al. 2020 [24]	1.68 (1.45–1.87)	0.037
Zhang L. et al. 2019 [25]	1.11 (1.04–1.20)	<0.01

OR—odds ratio; PWV—pulse wave velocity.

## Data Availability

Not applicable.
